# Outcome of a de-labelling algorithm compared with results of penicillin (β-lactam) allergy testing

**DOI:** 10.1186/s13223-022-00659-1

**Published:** 2022-03-22

**Authors:** Philipp Schrüfer, Johanna Stoevesandt, Axel Trautmann

**Affiliations:** Department of Dermatology and Allergy, Allergy Center Mainfranken, University Hospital Würzburg, 97080 Würzburg, Germany

**Keywords:** Anaphylaxis, Drug adverse reaction, Drug allergy, Drug exanthema, Drug hypersensitivity, Penicillin allergy, Penicillin hypersensitivity

## Abstract

**Background:**

Penicillin allergy labels frequently impede guideline-directed treatment with a penicillin or other β-lactam antibiotics. Despite presumed allergy, targeted questioning may indicate a low probability of sensitization and permit reasonably safe administration of the antibiotic in question. In this study, we evaluated a standardized algorithm aiming to differentiate non-allergic patients from those with true allergic β-lactam hypersensitivity.

**Methods:**

We retrospectively applied a de-labelling algorithm in 800 consecutive patients with suspected β-lactam hypersensitivity. All had undergone complete allergy work-up permitting to definitely exclude or diagnose β-lactam allergy between 2009 and 2019.

**Results:**

In 595 (74.4%) out of 800 cases evaluated, β-lactam allergy could be excluded by negative challenge testing. IgE-mediated anaphylaxis was diagnosed in 70 (8.7%) patients, delayed-type hypersensitivity in 135 (16.9%). In 62 (88.6%) anaphylaxis cases, the algorithm correctly advised to use an alternative antibiotic. Accuracy was higher in patients with moderate to severe anaphylaxis (97.7%) compared to those with a history of mild reactions (73.1%). The algorithm correctly identified 122 (90.4%) patients with proven delayed-type hypersensitivity. It permitted de-labelling in 330 (55.5%) out of 595 patients with diagnostic exclusion of penicillin hypersensitivity, but failed to identify the remaining 265 (44.5%) as low-risk cases.

**Conclusions:**

The algorithm detected 89.8% of cases with penicillin (β-lactam) allergy, sensitivity was optimal for moderate to severe anaphylaxis. Study data justify the implementation of a standardized de-labelling algorithm under close supervision in order to permit guideline-directed treatment and reduce the use of broad-spectrum antibiotics as part of an antibiotic stewardship program.

**Supplementary Information:**

The online version contains supplementary material available at 10.1186/s13223-022-00659-1.

## Background

Up to 10% of the population in Europe, North America, and Australia report penicillin allergy [1, 2]. Due to a label of penicillin allergy, the treating physician commonly feels compelled to administer an alternative antibiotic, even if a penicillin would be the treatment of choice. We are not aware of exact data concerning the question whether physicians refrain from prescribing the whole class of β-lactam antibiotics because of a vague report of penicillin allergy or avoid only penicillins, e.g. benzyl penicillin, phenoxymethyl penicillin, and aminopenicillins. In Germany, in our experience, all β-lactam antibiotics are usually avoided because of feared cross-reactivity, whereas in other countries a 2nd or 3rd generation cephalosporin may be used in such a situation. However, penicillin allergy labels increase the use of broad-spectrum antibiotics, which may further promote the problem of bacterial resistance [3–5]. In recent years, increasing attempts have been made in centers of allergy or infectious disease worldwide to critically question presumed penicillin allergy and, if necessary, directly de-label without testing via medical reconciliation [6–13]. De-labelling unproven penicillin allergy is increasingly important in the fight against antibiotic resistance [3, 14, 15].

Allergic hypersensitivity to penicillins or other β-lactam antibiotics most commonly manifests either within a few minutes after intake or infusion as acute anaphylaxis or several hours to days later as measles-like (maculopapular) exanthema; other reaction patterns are less common [1, 16–18]. A German retrospective observational study revealed aminopenicillins to most commonly cause exanthematous delayed-type reactions, whereas IgE-mediated anaphylaxis was predominantly attributed to certain cephalosporins, e.g. cefazolin, ceftriaxone or cefuroxime [19]. The cephalosporins mentioned are often administered intraoperatively and were identified as the most important trigger of an anaphylactic incident during general anesthesia by some authors [20, 21]. These observations, however, are dependent on prescription behavior and may vary among different countries.

This study is based on a group of 800 patients with suspected β-lactam allergy, all of whom underwent standardized allergy testing including diagnostic challenge [19]. The medical history of these cases was retrospectively reviewed and critically evaluated using a de-labelling algorithm. The outcome of the algorithm was then compared with the respective results of allergy testing.

## Methods

### Patients

The medical history of 800 consecutive patients referred to our allergy clinic from January 2009 to December 2019 for diagnostic work-up of a hypersensitivity reaction attributed to a β-lactam antibiotic was evaluated. The institutional review board of the University Hospital Würzburg consented to retrospective review and publication of anonymized data.

### Allergy testing

Allergy testing including β-lactam-specific serum IgE, patch, prick, and intradermal skin test, as well as challenge testing was performed as described previously in detail [19]. The severity of anaphylaxis was classified as mild, moderate or severe [22].

### De-labelling algorithm

**Fig. 1:**
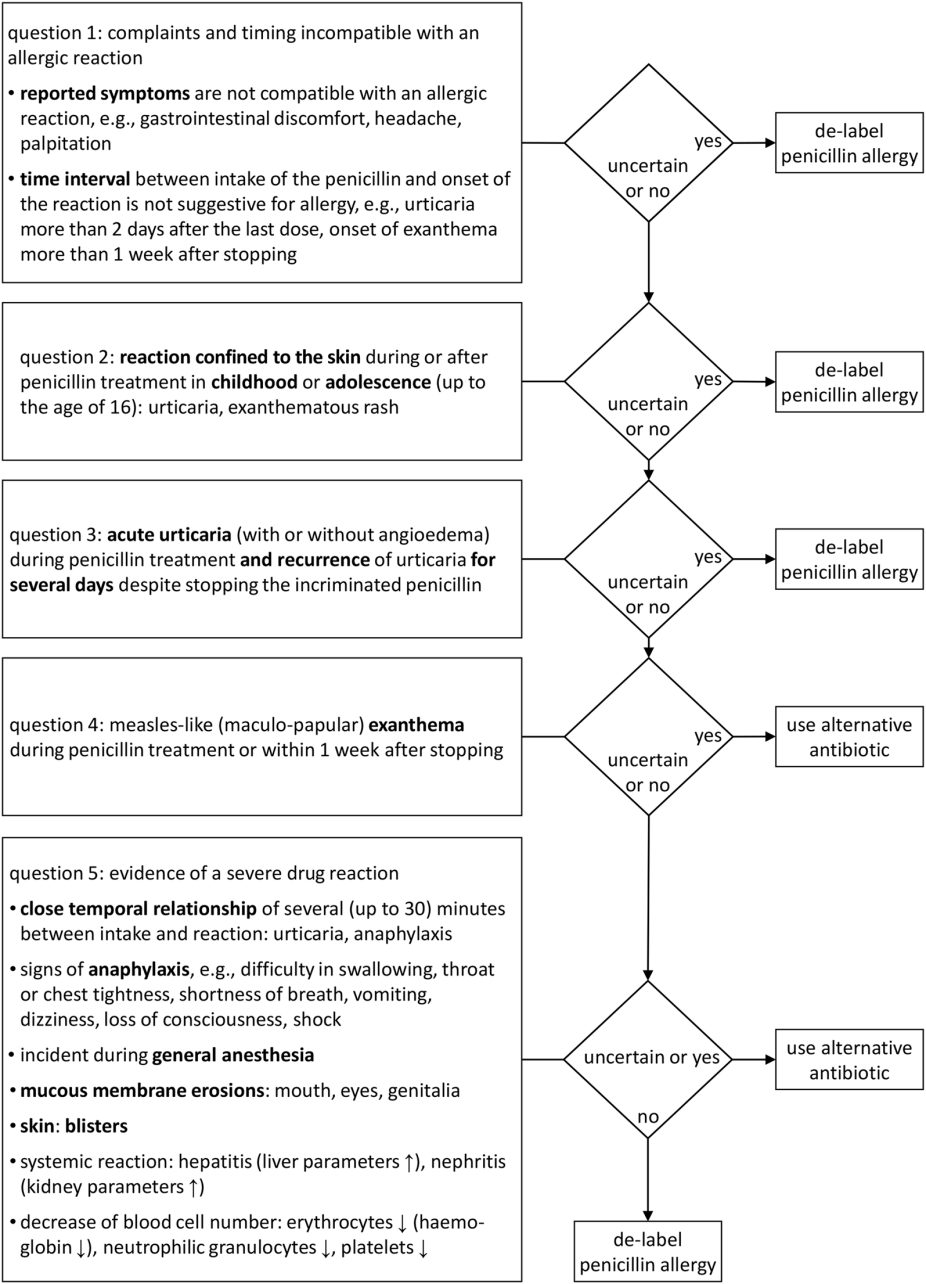
De-labelling algorithm applied to the medical history of 800 cases with suspected penicillin allergy [modified from (23)]

In a blinded approach, results of allergy testing were initially unknown to the investigator, and only patient’s medical history was evaluated using a modified version of our recently published de-labelling algorithm comprising five key questions (Fig. 1) [23]. The algorithm's outcome is binary, recommending either to de-label penicillin allergy (and administer the β-lactam in question) or to use an alternative antibiotic. Structure and content are briefly explained hereinafter, a critical review is included in the discussion section. Question 1 is intended to identify cases that are not indicative of allergic hypersensitivity because clinical complaints are incompatible or the time interval between intake and symptoms is clearly to long for an allergic reaction. Question 2 aims at skin reactions in childhood or adolescence without additional systemic symptoms, for which allergic penicillin hypersensitivity can be virtually excluded. Question 3 asks for a prolonged episode of urticaria without any signs suggestive of systemic anaphylaxis. Question 4 is targeted at the most common form of allergic penicillin hypersensitivity, a measles-like (maculopapular) exanthema in timely relationship with intake or administration. If the answer to questions 1-4 is either no or uncertain, question 5 needs to be addressed in order to exclude or identify potential indicators of a severe drug reaction. In addition to a close temporal relationship of only a few minutes between intake and clinical reaction, these include signs of systemic anaphylaxis, an incident during anesthesia, erosions of mucous membranes, cutaneous blisters, hepatitis, nephritis, or a sudden drop of blood cell numbers.

## Results

The reported type of hypersensitivity reaction, the incriminated β-lactam antibiotic, and the time interval between the β-lactam-associated reaction and allergy testing in all 800 cases is shown in Table 1. In 334 cases (41.8%), patient’s history was suggestive of an immediate reaction, in 421 (52.6%) of a delayed reaction. The assignment of the remembered incident as immediate or delayed remained uncertain in 45 patients (5.6%), 30 of whom reported a time interval of more than 10 years since the β-lactam-associated reaction.

**Table 1:** Patient history (data from allergist directed testing): type of hypersensitivity reaction, incriminated β-lactam antibiotic, route of intake or administration, and time interval between the β-lactam-associated reaction and allergy testing in 800 patients with suspected β-lactam hypersensitivity

	Immediate reaction (n = 334)	Delayed reaction (n = 421)	Assignment uncertain (n = 45)
Culprit β-lactam antibiotic			
Aminopenicillin (amoxicillin or ampicillin)	111	287	13
Cephalosporin	144	47	3
Benzyl/phenoxymethyl penicillin	45	55	9
Other	4	2	0
Unclear or insufficiently documented	30	30	20
Route of intake or administration			
Oral	235	346	40
Intravenous	97	75	2
Intramuscular	1	0	1
Unclear or not sufficiently documented	1	0	2
Time interval between β-lactam-associated reaction and allergy testing			
≤ 1 year	214	274	3
> 1–5 years	31	46	2
> 5–10 years	13	14	3
> 10 years	71	80	30
Unclear or insufficiently documented	5	7	7

### Results of allergy testing

In 595 (74.4%) cases, negative challenge testing finally excluded β-lactam hypersensitivity. The remaining 205 patients with proven β-lactam hypersensitivity were recently described in detail (Additional file 1) [19]. The diagnosis of delayed-type β-lactam hypersensitivity in 135 (16.9%) patients and IgE-mediated allergy in 70 (8.7%) was based on an overall assessment including history, reaction pattern, and results of testing.

### Outcome of the algorithm

**Fig. 2:**
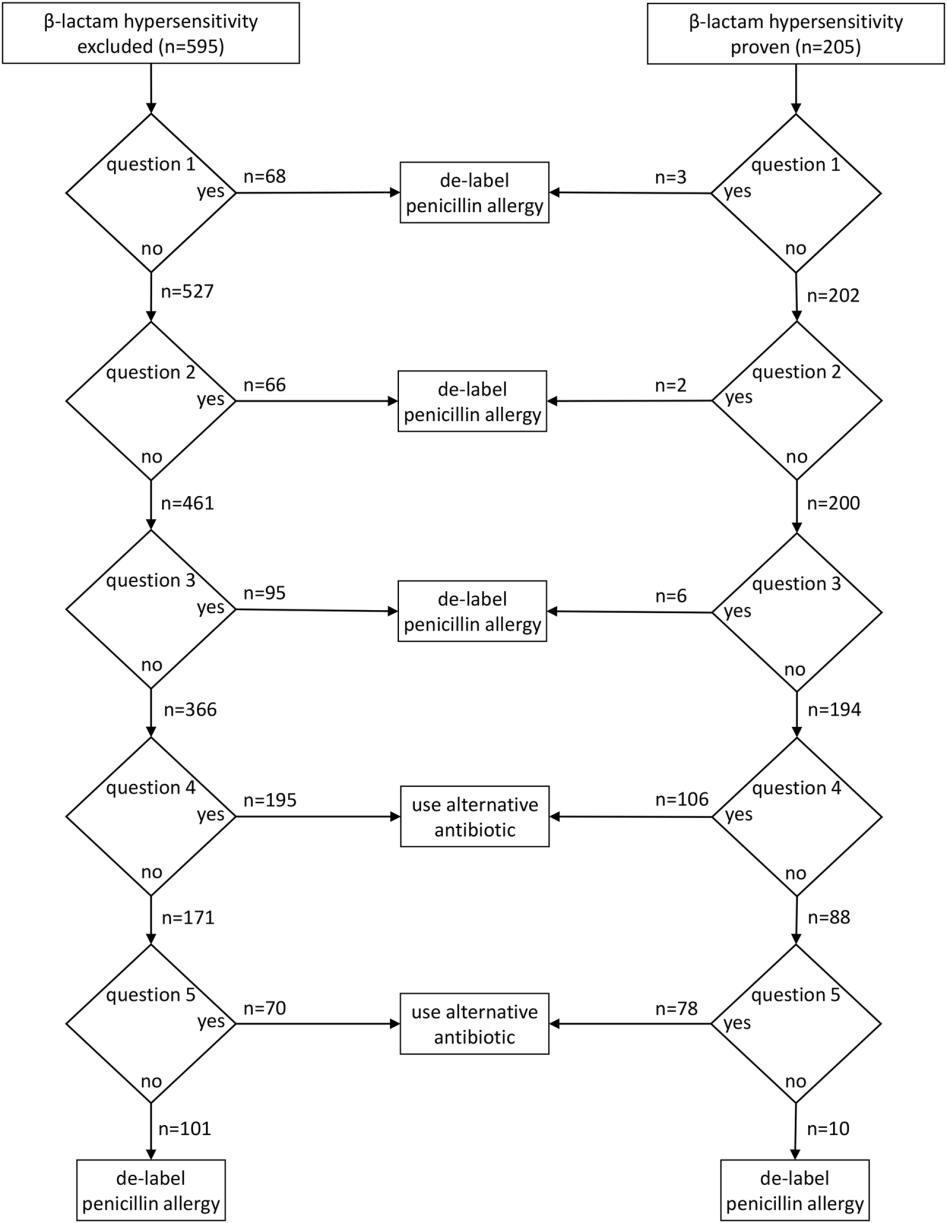
Outcome of the de-labelling algorithm applied to the medical history of 800 consecutive cases with suspected penicillin allergy. In 595 cases, β-lactam hypersensitivity could be definitely excluded through negative challenge testing, allergic β-lactam hypersensitivity was proven in 205 cases by positive results of skin or challenge testing

The algorithm’s outcome in all 800 cases is summarized in Fig. 2, comparing the results of patients with confirmed β-lactam allergy (n = 205) to those of non-allergic patients (n = 595). The algorithm recommended de-labelling for 21 (10.2%) patients with confirmed penicillin allergy—in real life this might have entailed re-administration of the antibiotic in question and thus an allergic reaction. On the other hand, the algorithm resulted in a recommendation to use an alternative antibiotic for 265 (44.5%) out of 595 non-allergic patients.

### Comparison between results of allergy testing and outcome of the de-labelling algorithm

The results of allergy testing and outcome of the de-labelling algorithm are depicted in Table 2. Out of 205 cases with proven allergic penicillin hypersensitivity, the algorithm would have correctly identified 184 (89.8%) and recommended use of an alternative antibiotic. Of note, 43 out of 44 cases (97.7%) with moderate to severe anaphylaxis were correctly detected. The three patients suffering from FDE and the three DRESS patients were identified as high risk cases by question 5 due to a respective history of cutaneous blistering or hepatitis. The algorithm correctly recommended use of an alternative antibiotic in 106 out of 117 cases (90.6%) with measles-like (maculopapular) exanthema as detected by question 4. The 21 cases of incorrect de-labelling comprised 8 anaphylaxis cases (7× mild, 1× moderate) and 13 delayed-type reactions (11× measles-like exanthema, 2× SDRIFE) (Fig. 2). The algorithm correctly advised de-labelling for 330 (55.5%) out of 595 non-allergic patients, but an alternative antibiotic would have been unnecessarily recommended for the remaining 265 (44.5%). The false suspicion of penicillin allergy was mainly attributed to a history of exanthema (195 cases) and to complaints that were incorrectly interpreted as signs of a severe drug reaction (70 cases).

**Table 2:** Comparison of results of allergy testing and outcome of the de-labelling algorithm

Allergy testing (data from allergist directed testing)	Outcome of de-labelling algorithm (blinded investigator assessment)		Sum
	De-labelling	Use alternative antibiotic	
β-lactam hypersensitivity excluded	330 (55.5%)	265 (44.5%)	595 (100%)
Allergic β-lactam hypersensitivity proven (any type)	21 (10.2%)	184 (89.8%)	205 (100%)
Immediate-type (anaphylaxis)	8 (11.4%)	62 (88.6%)	70 (100%)
Mild	7	19	26
Moderate	1	26	27
Severe	0	17	17
Delayed-type	13 (9.6%)	122 (90.4%)	135 (100%)
Measles-like (maculopapular) exanthema	11	106	117
SDRIFE	2	10	12
FDE	0	3	3
DRESS	0	3	3

*DRESS* drug reaction with eosinophilia and systemic symptoms, *FDE* fixed drug eruption, *SDRIFE* symmetrical drug related intertriginous and flexural exanthema

## Discussion

The majority of patients with a penicillin allergy label have never been tested and suspected penicillin hypersensitivity remains unproven. Study data show that our standardized algorithm is a useful tool for estimating the probability of true allergic hypersensitivity, which in many cases is so low that re-administration of the β-lactam antibiotic in question may be considered sufficiently safe. Application of the algorithm is, of course, restricted to patients who are not cognitively impaired, understand the questions and are able to answer rationally. As long as evidence of safety from large prospective studies is missing, use of the algorithm should be restricted to inpatients who subsequently receive the β-lactam antibiotic in question under close medical supervision.

The capacity of allergy care in Germany and elsewhere is limited, and the large number of cases—up to 10% of the European, North American, and Australian population report a history of penicillin allergy—makes testing of all patients virtually impossible [24]. To address this problem, centers of infectious disease or allergy around the world recently developed strategies to critically question penicillin allergy labels and, if necessary, de-label and re-administer the respective antibiotic directly without testing [6–10]. Following a different approach, some groups advocate allergy testing directly before initiation of treatment, meaning that the β-lactam antibiotic in question may only administered if skin testing is negative at 15 minutes reading [25–27]. Though this strategy might permit to identify cases of IgE-mediated anaphylaxis, it does not allow exclusion of delayed-type hypersensitivity. Moreover, routine implementation of standardized allergy testing prior to antibiotic therapy is hardly realistic in practices and emergency departments due to the lack of specialized staff, equipment, and—last but not least—time.

Question 1 of the presented algorithm aims to identify reports of non-specific complaints or pharmacological side effects that have mistakenly lead to a suspicion of allergic hypersensitivity. This does include abdominal pain, palpitation, or headache—symptoms that do not indicate an allergic reaction on their own, even if interpreted as such by the patient and/or treating physician [1]. Moreover, a prolonged time interval between last intake and the onset of symptoms (>2 days to onset of urticaria, >1 week to onset of exanthema) strongly speaks against allergic hypersensitivity, provided that the patient is able to reliably recall the time course. Question 2 addresses an urticarial or exanthematous skin rash in childhood or adolescence without any further complaints which almost always results from a bacterial or viral infection and is only rarely caused by allergic hypersensitivity [28–30]. Penicillin-induced anaphylaxis is unlikely if medical history reveals urticaria without further systemic symptoms of e.g. respiratory tract or cardiovascular system, especially if episodes of urticaria recur several days after stopping penicillin treatment (question 3) [31]. In case of an acute urticaria episode directly after intake of a penicillin, users of the algorithm will be guided to question 5 in order to assess the risk of systemic anaphylaxis. Questions 1-3 permitted correct de-labelling in 229 non-allergic patients (question 1: n = 68, question 2: n = 66, and question 3: n = 95) while proving sufficiently safe. An incorrect recommendation to de-label which might have lead to re-administration of the respective antibiotic and thus an allergic reaction in a real life situation was given in only 11 cases (question 1: n = 3, question 2: n = 2, and question 3: n = 6) (Fig. 2).

Penicillin should be preferably avoided if question 4 is answered with yes, provided that the exanthematous rash developed during adulthood and not in childhood as addressed in question 2. Measles-like (maculopapular) exanthema generally is not a severe drug reaction (16, 32). As a consequence, the recommendation to switch to an alternative antibiotic is rather on the cautious side. Accordingly, in other published pathways exanthema is not taken into account at all [10]. Our data show that in 195 (32.8%) out of 595 non-allergic patients, an alternative antibiotic would have been recommended unnecessarily due to question 4 (Fig. 2). On the other hand, by the same question 4 recurrence of exanthema would have been prevented in 106 (51.7%) out of 205 patients with proven penicillin allergy. This type of drug reaction should not be trivialized but considered as bothersome and sometimes protracted condition, which represents an additional and potentially preventable burden for a patient already suffering from an infectious disease [32].

If none of the previous questions 1–4 could be unequivocally answered with yes, the user will be guided to question 5 addressing evidence of a severe drug reaction. Anaphylaxis usually develops within a few minutes after intake of the drug, presenting as a systemic reaction including sudden cardiovascular (e.g. arterial hypotension, tachycardia, loss of consciousness) and/or respiratory symptoms (e.g. cough, chest tightness, shortness of breath). As administration of an antibiotic is nowadays considered the most common cause of intraoperative anaphylaxis [20, 21], the algorithm specifically addresses incidents during general anesthesia. Signs of a severe drug-induced delayed reaction include painful erosions of the oral mucosa and/or cutaneous blisters, but also hepatitis, nephritis or a sudden drop of cell numbers in peripheral blood [33]. Evaluation of question 5 underlined the difficulties and limitations arising from self-reported information with regard to both exclusion and reliable detection of a potentially severe drug reaction. Based on retrospective interpretation of reported symptoms as potentially severe, an alternative antibiotic would have been unnecessarily recommended by question 5 in 70 out of 171 non-allergic patients (Fig. 2). Eighty-eight out of 205 patients with proven penicillin allergy were evaluated according to question 5; in 78 a recommendation to use an alternative antibiotic was correctly given, whereas de-labelling was recommended for the remaining 10 who probably would have developed an allergic reaction upon re-exposure of the respective antibiotic.

The PEN-FAST was recently proposed as an even more straightforward approach for de-labelling questionable penicillin allergy [10]. PEN-FAST represents a clinical decision rule consisting of five short questions resulting in a point score [PEN: penicillin allergy reported by patient (if yes, proceed with assessment), F: five years or less since reaction (2 points), A: anaphylaxis or angioedema (2 points), S: severe cutaneous adverse reaction (2 points), T: treatment required for the reaction (1 point)] [10]. A total score of less than three points was determined as cutoff value for a low risk of penicillin allergy (and thus de-labelling), whereas three or more points may indicate a higher risk (and thus administration of an alternative antibiotic). In a cohort of 622 patients, the calculated sensitivity of the PEN-FAST applying the mentioned cutoff value to identify penicillin allergy was 70.7%, specificity 78.5%, the positive predictive value was 25.3%, and the negative predictive value 96.3% [10]. The corresponding findings applying the presented algorithm in our series was a sensitivity of 89.8%, a specificity of 55.5%, a positive predictive value of 41.0%, and a negative predictive value of 94.0% (Table 2; Additional file 2). The results of these studies are not directly comparable mainly because of different populations investigated, e.g. the PEN-FAST study of total 622 cases included 58 with a positive finding in penicillin allergy testing (9.3%), whereas in our study we evaluated 800 cases including 205 with proven β-lactam hypersensitivity (25.6%). However, both de-labelling procedures seem to be quite safe as demonstrated by a high negative predictive value of >90%.

In addition to the presented data from a series of retrospective cases, a preliminary prospective study of 200 patients has demonstrated that the algorithm may be reasonably safe [23]. Data from both studies, however, demonstrate, that use of the proposed algorithm does not guarantee absolute safety. As in any other medical procedure, both the treating physician and the patient will be obliged to accept a certain residual risk which needs to be weighed against the benefits of guideline-directed antibiotic treatment.

### Reason for this study

Due to the limited number of patients with true penicillin allergy, sufficiently large prospective studies investigating the practicability, predictive value, and safety of the proposed de-labelling algorithm are missing to date. Until more robust data are available, well-studied patient series are a viable option to estimate the effectiveness and safety of such a de-labelling procedure.

### Limitations of our study

Data were retrospectively extracted from patient records, resulting in a certain inhomogeneity. As a consequence, we limited ourselves to a descriptive presentation of data. In this series, no cases of a severe bullous skin reaction of the SJS-TEN spectrum were included.

## Conclusions

(i) Up to 10% of the population in Europe, North America, and Australia report penicillin allergy and most of these cases are not verified by allergy diagnostics. A label of penicillin allergy does not automatically require administration of an alternative antibiotic. Physicians should preferably estimate the probability of allergic hypersensitivity by standardized questioning.
(ii) The algorithm applied in this study permits to detect evidence of allergic penicillin hypersensitivity with sufficient reliability, especially in cases of moderate to severe anaphylaxis.
(iii) The algorithm-based recommendation to use an alternative antibiotic for patients reporting measles-like (maculopapular) exanthema should be critically reconsidered as (i) allergic hypersensitivity could be excluded by allergy testing in a significant number of cases, and (ii) measles-like exanthema is not a severe drug reaction to be avoided at all costs.
(iv) Prospective studies of a sufficient size will be required to confirm the efficacy and safety of the proposed algorithm. Until more robust data are available, use of the algorithm should be confined to inpatients subsequently taking or receiving the penicillin or cephalosporin under direct medical supervision.

## Supplementary Information

**Additional file 1: **Diagnosis and sensitization to different β-lactams in 205 patients with allergic hypersensitivity (data from allergist directed testing) (modified from [19]).

**Additional file 2: **Two X Two table depicting results of allergy testing and outcome of the de-labelling algorithm in total 800 patients.

## Data Availability

Not applicable (this is a retrospective data evaluation).

## Abbreviations

DRESS: Drug reaction with eosinophilia and systemic symptoms
FDE: Fixed drug eruption
SDRIFE: Symmetrical drug related intertriginous and flexural exanthema
