# Supplementary material for: Outcome of a de-labelling algorithm compared with results of penicillin (β-lactam) allergy testing
Source: Allergy Asthma Clin Immunol. 2022 Mar 22;18:26. doi: 10.1186/s13223-022-00659-1 (PMC8941741; doi:10.1186/s13223-022-00659-1)
Supplement: Supplementary file 1 — Additional file 1: Diagnosis and sensitization to different β-lactams in 205 patients with allergic hypersensitivity (data from allergist directed testing) (modified from [19]). [file 13223_2022_659_MOESM1_ESM.doc]

# Additional file 1. Diagnosis and sensitization to different β-lactams in 205 patients with allergic hypersensitivity (data from allergist directed testing) [modified from (19)].

|  | | immediate-type hypersensitivity (n=70) | delayed-type hypersensitivity (n=135) |
| --- | --- | --- | --- |
| anaphylaxis | mild | 26 | n.a. |
|  | moderate | 27 |  |
|  | severe | 17 |  |
| delayed reaction | measles-like exanthema | n.a. | 117 |
|  | SDRIFE |  | 12 |
|  | FDE |  | 3 |
|  | DRESS |  | 3 |
| sensitization to β-lactam(s) confirmed by allergy testing | |  |  |
| aminopenicillin (amoxicillin and ampicillin) | | 3 | 91 |
| aminopenicillin and benzyl penicillin | | 2 | 36 |
| cephalosporin | | 47 | 4 |
| cephalosporin and aminopenicillin | | 12 | 0 |
| benzyl / phenoxymethyl penicillin | | 3 | 3 |
| piperacillin/tazobactam | | 3 | 0 |
| flucloxacillin | | 0 | 1 |

DRESS, drug reaction with eosinophilia and systemic symptoms; FDE, fixed drug eruption; SDRIFE, symmetrical drug related intertriginous and flexural exanthema; n.a., not applicable
