# Supplementary material for: Outcome of a de-labelling algorithm compared with results of penicillin (β-lactam) allergy testing
Source: Allergy Asthma Clin Immunol. 2022 Mar 22;18:26. doi: 10.1186/s13223-022-00659-1 (PMC8941741; doi:10.1186/s13223-022-00659-1)
Supplement: Supplementary file 2 — Additional file 2: Two X Two table depicting results of allergy testing and outcome of the de-labelling algorithm in total 800 patients. [file 13223_2022_659_MOESM2_ESM.doc]

# Additional file 2. Two X Two table depicting results of allergy testing and outcome of the de-labelling algorithm in total 800 patients.

|  | outcome of de-labelling algorithm | |  |
| --- | --- | --- | --- |
| results of allergy testing | de-labelling (i.e. **low risk of β-lactam hypersensitivity**) | use alternative antibiotic (i.e. **high risk of β-lactam hypersensitivity**) | sum |
| **β-lactam hypersensitivity excluded** | 330 | 265 | 595 |
| **allergic β-lactam hypersensitivity proven** | 21 | 184 | 205 |
| sum | 351 | 449 | 800 |
